# *“They talked to me rudely”.* Women perspectives on quality of post-abortion care in public health facilities in Kenya

**DOI:** 10.1186/s12978-023-01580-5

**Published:** 2023-02-27

**Authors:** Ramatou Ouedraogo, Grace Kimemia, Emmy Kageha Igonya, Sherine Athero, Shelmith Wanjiru, Martin Bangha, Kenneth Juma

**Affiliations:** African Population Health and Research Center, APHRC Campus, Manga Close, Kitisuru, P.O. Box 10787, Nairobi, 00100 Kenya

**Keywords:** Post-abortion care, Quality of care, Patients, Stigmatization, Kenya, Soins post-avortement, Qualité des soins, Patientes, Stigmatisation, Kenya

## Abstract

**Background:**

Access to safe abortion is legally restricted in Kenya. Therefore, majority women seeking abortion services in such restrictive contexts resort to unsafe methods and procedures that result in complications that often require treatment in health facilities. Most women with abortion-related complications end up in public health facilities. Nevertheless, evidence is limited on the quality of care provided to patients with abortion complications in public health facilities in Kenya.

**Methods:**

Data for this paper are drawn from a qualitative study that included interviews with 66 women who received post-abortion care in a sample of primary, secondary and tertiary public health facilities in Kenya between November 2018 and February 2019. The interviews focused on mechanisms of decision-making while seeking post-abortion care services, care pathways within facilities, and perceptions of patients on quality of care received including respect, privacy, confidentiality, communication and stigma.

**Findings:**

The participants’ perceptions of the quality of care were characterized as either “bad care” or “good care”, with the good care focusing on interpersonal aspects such as friendliness, respect, empathy, short waiting time before receiving services, as well as the physical or functional aspects of care such as resolution of morbidity and absence of death. Majority of participants initially reported that they received “good care” because they left the facility with their medical problem resolved. However, when probed, about half of them reported delays in receiving care despite their condition being an emergency (i.e., severe bleeding and pain). Participants also reported instances of abuse (verbal and sexual) or absence of privacy during care and inadequate involvement in decisions around the nature and type of care they received. Our findings also suggest that healthcare providers treated patients differently based on their attributes (spontaneous versus induced abortion, single versus married, young versus older). For instance, women who experienced miscarriages reported supportive care whereas women suspected to have induced their abortions felt stigmatized.

**Conclusion:**

These findings have far reaching implications on efforts to improve uptake of post-abortion care, care seeking behaviors and on how to assess quality of abortion care. There should be emphasis on interventions meant to enhance processes and structural indicators of post-abortion care services meant to improve patients’ experiences throughout the care process. Moreover, more efforts are needed to advance the tools and approaches for assessing women experiences during post-abortion care beyond just the overriding clinical outcomes of care.

**Supplementary Information:**

The online version contains supplementary material available at 10.1186/s12978-023-01580-5.

## Plain language summary

Access to abortion is legally restricted and socially reproved in Kenya. Therefore, women requiring abortion in such restrictive contexts resort to unsafe methods that result in complications, often requiring treatment in health facilities. Nevertheless, there is limited evidence on the quality of care provided in public health facilities in Kenya to patients treated for abortion complications. This paper is drawn from a qualitative study targeting 66 women treated for abortion complication in a sample of primary, secondary and tertiary public health facilities in Kenya between November 2018 and February 2019. The interviews focused on the women’s perceptions around the quality of care they received.

Our findings show that while the majority of participants stated in first instances that they received “good care” because they left the facility with their medical problem resolved, half of them, when probed, reported delays in receiving care, yet their condition was seen as an emergency since they were bleeding and experiencing pain. Participants also reported instances of abuse (verbal and physical) or lack of privacy during care and inadequate involvement in decisions on the type of care they were to receive. Our findings also point out that providers treated patients differently based on their attributes (spontaneous versus induced abortion, single versus married, young versus older), with women who experienced miscarriages receiving supportive care while women suspected to have induced their abortion being stigmatized.

In conclusion, our findings have far reaching implications on efforts to improve post-abortion seeking behaviors and on how to assess quality of abortion care.

**Supplementary Information:**

The online version contains supplementary material available at 10.1186/s12978-023-01580-5.

## Résumé

**Contexte:**

L’accès à l’avortement sécurisé est légalement restreint au Kenya. Par conséquent, la majorité des femmes en quête de services d'avortement ont recours à des méthodes et procédures dangereuses qui entraînent des complications nécessitant souvent un traitement dans des établissements de santé, bien souvent publics. Néanmoins, les données sont limitées quant à la qualité des soins offerts aux patientes souffrant de complications liées à l'avortement dans les établissements publics de santé au Kenya.

**Méthodes:**

Les données de cet article sont tirées d'une étude qualitative réalisée entre novembre 2018 et février 2019 à partir d’entretiens approfondis avec 66 femmes ayant reçu des soins post-avortement dans un échantillon d'établissements de santé publics de niveau primaire, secondaire et tertiaire au Kenya. Les entretiens ont porté sur les mécanismes de prise de décision lors de la recherche de services de soins post-avortement, les parcours de soins au sein des établissements, et les perceptions des patientes sur la qualité des soins reçus, notamment le respect, l'intimité, la confidentialité, la communication et la stigmatisation.

**Résultats:**

Les perceptions des participantes sur la qualité des soins ont été caractérisées comme « mauvais soins » ou « bons soins », les bons soins portant sur des aspects interpersonnels tels que l'amabilité, le respect, l’empathie, un temps d'attente court avant de recevoir des services, ainsi que sur les aspects physiques ou fonctionnels des soins tels que la disparition de la morbidité et l'absence de décès. La majorité des participantes ont dans un premier temps déclaré avoir reçu de « bons soins » car elles ont quitté l'établissement avec leurs problèmes médicaux résolus. Cependant, lorsqu’on les a relancées, environ la moitié d’entre elles ont signalé des retards dans leur prise en charge malgré l’urgence de leur état (c’est-à-dire des saignements et des douleurs graves). Les participants ont également signalé des cas de violence (verbale et sexuelle) ou d’absence d’intimité pendant les soins, ainsi qu'une faible implication dans les décisions concernant la nature et le type de soins qu'elles devaient recevoir. Nos résultats suggèrent également que les soignants ont traité les patientes différemment en fonction de leurs attributs (avortement spontané ou provoqué, célibataire ou marié, jeune ou âgé). Par exemple, les femmes qui ont eu un avortement spontané ont rapporté avoir bénéficié de soutien, tandis que celles soupçonnées d'avoir provoqué leur avortement se sont senties stigmatisées.

**Conclusion:**

Ces résultats ont des implications importantes dans les efforts visant à améliorer le recours aux soins post-avortement, les pratiques de recours aux soins et la manière d'évaluer la qualité des soins liés à l’avortement. Il convient de mettre l’accent sur les interventions destinées à améliorer les processus et les indicateurs structurels des services de soins post-avortement afin d’améliorer les expériences des patientes tout au long du processus de soins. En outre, des efforts supplémentaires sont nécessaires pour faire progresser les outils et les approches permettant d'évaluer les expériences des femmes pendant les soins post-avortement au-delà des seuls paramètres cliniques dominants des soins.

## Introduction

Kenya is one of the countries known for having restrictive abortion laws in Africa. According to article 26 of the 2010 Constitution, access to safe abortion is only permitted when “in the opinion of a trained health professional, there is a need for emergency treatment, or the life or health of the mother is in danger, or if permitted by any other written law” [1]. As a result, women seeking abortion services and providers involved in provision of abortion care risk heavy fines and criminal prosecutions, including imprisonment. This legal context, combined with the stigma around abortion, means that the bulk of women in need of abortion services resort to unsafe methods and procedures. In 2012, an estimated 464,000 induced abortions occurred in Kenya, the majority of which were unsafe [2]. More than 30% of these abortion resulted in complications that required intensive care and attendance by highly-skilled health providers [3, 4].

While access to safe abortion is restricted in much of Sub-Saharan Africa (SSA), several countries including Kenya have committed to address abortion-related maternal morbidity and mortality by availing post-abortion care (PAC) services to all women [5, 6]. PAC is a set of interventions that include treatment of complications, provision of contraceptive counseling and services, counseling to address emotional and physical needs, referral, and partnership with the community [7]. In 2012, the Kenyan Ministry of Health launched the “*Standards and Guidelines for Reduction of Maternal Morbidity and Mortality from Unsafe abortion”* in an effort to improve the quality of care for women after terminating a pregnancy. However, these guidelines were later withdrawn by the Ministry of Health, and then reinstated by a High court ruling in 2019.

Despite these efforts, very few women are able to access PAC services within healthcare facilities [8]. A systematic review by Izugbara et al. (2019) identified a range of complex socioeconomic, infrastructural, cultural and political factors that impede availability, accessibility and utilization of PAC services in SSA, including limited capacity of health facilities to provide PAC due to unavailability of staff and absence of PAC equipment and supplies [9]. Increasingly, studies assessing patients' experiences with PAC services offered in healthcare facilities have reported high levels of patient satisfaction [10, 11]. Further, studies have explored how patients' perceptions of the quality of care is closely linked to patient attributes, providers attitudes, and the capacity of health facilities to deliver services [11, 12]. However, there have been very few studies that have examined patients' perceptions on the quality of PAC in Kenya. So far, only a single study focused on the quality of pregnancy termination services within private health facilities [13]. Yet, such evidence is key in designing or implementing programs that aim to improve quality of care, patient care experiences following an abortion complication, and by extension, enhancing uptake of post-PAC contraception. Indeed, there is evidence of high rates of repeat abortions among PAC patients in Kenya [2, 3, 13], which raises questions about the quality of PAC services in these facilities, particularly whether post-abortion counseling and contraception are provided.

This paper explores the perspectives of patients on the quality of PAC received in public health facilities throughout the care process. According to the World Health Organization (WHO), quality healthcare should be effective (evidence-based healthcare), safe (free from harm), and people-centered (responsive to individual preferences, needs and values). In recent decades, quality of care has become the cornerstone of health systems reforms [14], with significant investment towards strengthening health systems to meet users’ expectations and achieve their desired health outcomes [15]. However, the concept of quality of care is complex, highly subjective, and difficult to assess and measure. The complexity is based on the fact that quality care can be almost anything anyone wishes or wished to have had. To address this, Donabedian [16] developed one of the most widely used frameworks for assessing the quality of healthcare. Our paper draws on this framework to reflect on patients’ experience with the quality of post-abortion care. Donabedian’s tripod model assesses healthcare quality based on structures, processes and outcomes [16]. The structural domain defines the environment and context in which health care is provided, including service delivery facilities, staffing and training, equipment, technology, cost, and models of service delivery. The process of care refers to the methods by which healthcare is provided, including the interactions with providers, how the services are offered, and how this intersects with patients’ care expectations. Outcomes are defined as changes that occur in patients’ health status (whether immediate or future) that can be attributed to the healthcare provided. The Donabedian model argues that enhancement in structures of care will automatically contribute to improved patients outcomes. Therefore, when used appropriately, both process and outcome measures can provide useful information about the quality of care.

Building on the Donabedian model, this paper assesses women’s perspectives on the quality of post-abortion care they received in Kenyan public health facilities based on the process domain, regardless of the actual consequences or outcomes. Quality care, also regarded as “good care” by patients, is perceived during service delivery, and focuses on whether the process of care conforms to the practices expected to produce the best possible results. Even with the appropriate structures and processes in place, outcomes may or may not be achieved. Inversely, positive or negative outcomes do not necessarily imply good or bad care. In some cases, patients’ clinical condition may improve regardless of quality of care. As such, when women report satisfaction with care [10, 11], they may be doing so because of the positive health outcomes, while disregarding challenges in the process of care. Consequently, care processes form the basis for assessment of care quality utilizing known best practices and conceptualization of the relationship between care processes and attainment of desired outcomes, which is reflected in person centered care frameworks [17].

We focus on women’s perceptions of quality of care that entails processes of accessing care including interactions with providers, waiting time and cost, as well as how structural and normative aspects of PAC and abortion such as lack of training or equipment, or abortion stigma drives these processes. We examine how the women were treated at the facilities by health providers and the interpersonal elements such as privacy, confidentiality, involvement in decision-making, communication and empathy.

## Methods

### Study design and population

Data for this study were drawn from a large multi-country study on the quality of post-abortion care in public health facilities in Burkina Faso, Kenya and Nigeria, utilizing both quantitative and qualitative methods. In this paper, we only analyzed the qualitative study conducted in Kenya. We applied a phenomenology design to explore the lived experiences of women treated for abortion complications to assess the uniqueness of their experiences based on their attributes (i.e., being married or single, young or adult, the level of health facility where they were treated- primary or referral facilities). Data collection involved in-depth interviews (IDI) with patients who received PAC in a sample of primary (Level II and III) and referral facilities (Level IV, V and VI) across seven counties in Kenya, namely: Garissa, Kajiado, Kiambu, Laikipia, Mandera, Migori, and Nairobi. The interviews were complemented with direct observations of patients and providers interactions during PAC services delivery. Data collection took place between November 2018 and February 2019.

### Data collection process

We recruited six research assistants with backgrounds in sociology or anthropology and had previous experience conducting qualitative data collection. The research assistants were then taken through a five-day training that covered study objectives, aims of the qualitative study, target population, ethical considerations, and interviewing and observation techniques. They also participated in the pre-testing and review of the interview guides.

Within the targeted health facilities, women treated for abortion complications were purposively selected. Selection of participating women was based on their age, marital status, education level, occupation, and the level of the facility where they were treated. Prior to discharge, healthcare providers who deliver PAC introduced the patients to the study and research assistants stationed at the facilities, and only those who consented to participate were interviewed. The interviews took place within the health facilities in secluded spaces to ensure confidentiality. The interviews were conducted based on an interview guide that focused on exploring the decision-making processes to seek PAC, their pathways within the facilities, their perceptions of quality care, and how it informed their care-seeking pathways, as well as their experiences with the quality of PAC services received (for additional information, please refer to the guide—Additional file 1). While our initial target was to interview 50 patients, we increased the number to 66 participants to achieve saturation. Throughout the data collection period, the study team listened to the audios and had weekly debrief meetings to discuss emerging information and variation or stagnation in the experiences of women depending on their personal profiles and the facilities levels. The interviews were conducted in three local languages, namely Swahili, Luo, and Somali, and, with participants consent, they were audio-recorded. Participants’ socio-demographic characteristics are summarized in Table 1.

**Table 1:** Participants socio-demographic characteristics

Characteristic	Frequency (N = 66)
Age (Years)	
14–19	6
20–29	33
30 and above	26
Missing	1
Area of resident	
Urban	55
Rural	11
Marital status	
Married/cohabiting	43
Separated/divorced/widowed	2
Single	15
Missing	6
Occupation	
Employed	44
Unemployed	11
Missing	11

### Data analysis

The IDIs were transcribed and translated into English when needed. Data was analyzed by a team of researchers using thematic analysis approach. We first developed a codebook from the interview guide and by reviewing a few transcripts and field notes from each of the seven counties. The codebook was used to code a set of transcripts to ensure accuracy and capture any missing codes. The codebook was updated and the comprehensive codebook applied to all the transcripts and field notes using Nvivo version 12. For this paper, we selected and discussed emerging themes related to patients' perceptions of quality care (namely good and bad care), patients’ experiences using PAC including decision-making processes, time taken before receiving care, and their interaction with providers. More details on the emerging themes are provided in Additional file 2.

### Ethical considerations

The study protocol was reviewed and approved by the AMREF Ethics and Scientific Research Committee (ESRC) (protocol ID: AMREF-ESCR P429/2018), and the University of Nairobi/Kenyatta National Hospital Ethics and Research Committee (protocol ID: KNH-ERC/A/384) in Kenya. Permits to conduct the study were also obtained from the Kenyan National Commission for Science, Technology and Innovation (NACOSTI) and from each participating health facility. Individual written consent was obtained from every participant before their involvement in the study, including those below 18 years who were regarded as emancipated minors. Confidentiality, anonymity, and privacy of all participants were guaranteed at all levels of the study by excluding all unique identifiers for patients and access to study data was limited to the research team.

## Results

### Participant definition of quality care

Study participants shared their perceptions of what they considered as “good care” and/or “bad care” based on their previous experiences in health facilities. In their description of “good care” in the course of their interactions with service providers, we noted the frequent use of expressions such as friendliness/kindness, absence of harassment, respect, and empathy, as well as absence of long waiting times and low cost of care (affordable). Some participants considered good care as receiving the appropriate medical care or feeling that their health improved when leaving the facility, even if they had encountered challenges with providers. On the other hand, “bad care” was associated with harsh or rude providers, physical assault, long-waiting time, and the absence of empathy and proper communication, as explained by one of the PAC patients:

*For bad care, we used to feel that the doctors and nurses are harsh on how they talk to people. That is why I don’t go to a hospital, they are so rude, they can shout at you, they can even beat you because you did something, they don’t care how you feel. In good care, you feel your patients’ pain is your pain* (25-year-old, single, business lady, urban, Nairobi).

For the participants, their previous experiences in a health facility, not necessarily related to PAC, informed their decision to seek PAC services in particular facilities they visited. Notably, good experiences of previous care, the cost of service, and their financial resources influenced their choice of facility. This is explained by one of the participants:

*I wanted to come here because it is cheap, it’s not the same as other hospitals because it depends on your earnings, you can go to an expensive one or a cheap one. So I decided according to my means, I should be brought here* (23-year-old, single, unemployed, urban, Kajiado).

However, some participants did not experience the same good care when they went for PAC. Moreover other participants who visited primary-level facilities—based on their previous experiences of good care while seeking treatment for a different medical issue—ended up being referred because the facility chosen could not offer PAC services. This referral process delayed access to care for some of the participants. Indeed, since most primary-level facilities lacked ambulances to facilitate transport to higher-level facilities, some participants had to wait for satellite ambulances which are often managed by the sub-county administration and are stationed in other facilities. Ambulances were therefore not always available, and sometimes would take a long time to get to facilities located in remote and distant areas. In such situations, some participants were forced to use alternative means (i.e., public transport) to get to the referral facility, and they had to cover these additional costs incurred during the referral process.

### Time spent before receiving treatment

One aspect of care that participants considered while judging whether they experienced good or bad care, was the amount of time they spent in the health facility. Participants’ opinions were diverse regarding the amount of time it took for them to be attended to and receive full treatment, mainly Manual Vacuum Aspiration (MVA) or Medical Abortion (MA). Almost half of the participants reported being attended to immediately upon arrival, while others reported delays of between one to many days. Most participants who were treated immediately upon arrival were often emergency cases, such as those with heavy bleeding, or in severe pain, or those referred from other facilities. A participant from Laikipia described how fast she was attended to once she arrived and equated the speed of receiving care to “good” care:

*Actually it was an emergency so they started it on the spot and they were so nice to me, because even if they didn’t know what happened, because maybe I might even have done something like abortion or something, but they didn’t take it that way because it was an emergency. And later on that is when they asked me if I did anything or something. But, even after realizing that I did not do abortion or something, they did treat me well* (31-yearsold, married, teacher, urban, Laikipia)*.*

Unlike this patient, others who reported delays in receiving care also felt that their conditions were emergency cases, since they were heavily bleeding and in severe pain. However, they were forced to wait for long hours because of the long line of patients waiting to be attended to and few providers available to provide care. One participant, who bled excessively and ended up losing her pregnancy while still waiting in line, felt so embarrassed and disappointed. The participant felt that her case should have been assessed and prioritized during triage instead of making her to wait for long as she narrated:

*They should at least look at the state of the patient when they get here, observe them instead of making them queue and maybe they don’t know what their situation is; would they have checked my situation, I wouldn’t have had the problem. I bled while I was just sitting and had the abortion there. So had they attended to me fast when I came, because I was in pain, you know I would at least have bled there and I wouldn’t have bled in front of everyone else* (35-year-old, single, employed, urban, Kiambu).

A 23-year-old participant from Kajiado, who *“was told the child had died in the womb”,* felt that her situation was an emergency that required immediate attention. Instead she was kept waiting for long hours, a situation that exposed her to more pain and could have potentially worsened her condition.

Moreover, even among participants who expressed satisfaction with their waiting time at the facility, their perspectives were diverse. For instance, a number of participants persevered through the long lines before receiving care, but felt the waiting time was justifiable and reasonable because of the large number of patients in the queue, or sometimes the low number of service providers available. The narratives below are an illustration of those participants who felt waiting time was acceptable in relation to the number of patients and service providers available:

*I can say the patients were many, and some came ahead of me. There was a patient being attended to, so I could not blame the doctor because she was alone and the patients were many* (33-years-old, married, employed, urban, Kajiado).

*What I can say is that we have doctors but they are few; only one will work in all the departments, you have to wait for him to go there, come back to the maternity, see patients, so you come and wait for long* (35-year-old, married, self-employed, rural, Migori).

As illustrated by the participants above and many others, they felt that most healthcare providers would ideally like to provide immediate care to all patients in emergency situations. However, providers often struggle with the challenge of determining which emergency cases to prioritize, as they cannot attend to all patients simultaneously. Cases of providers’ burnout were also reported among the reasons for delays in services, particularly for patients who arrived at the facility in the evening. During our observation at one of the referral facilities, we observed a service provider taking a nap while patients were still waiting in the queue to be attended to. As a result, the number of patients waiting increased, and new emergencies developed because of the long waiting time. This was a common issue in facilities with a high volume of patients and few providers (i.e., referral level facilities).

In some cases, the referral process prolonged the time spent in health facilities. For instance, participants who had initially been admitted but were then referred for other services such as ultrasound scans, took longer to receive treatment. The following case demonstrates how referrals for ultrasound or other tests can delay the care and worsen patients’ conditions:

*…So when I got there I started bleeding, then I was told to go for an ultrasound. I took the stairs and went for that. I was then sent to go for a Rhesus test to know my blood group, I went and got tested. Then I was sent again to test for PDT, again I was tested (…). So, when I was there I kept using stairs and coming down, so I started feeling my abdomen being painful like I am in labor. So that’s when…because I had also drank a lot of water, I went down to the toilet and I was bleeding profusely and could see clots coming out; then I would go and wait for results then go back to the toilet and bleed. So, when I was given the results, I took them to the doctor down there and that’s where there was a problem, I waited for long to give him the results because people were so many and the doctors were very few. I waited for a long and the abdomen pain continued like labor* (36-yearold, married, casual laborer, urban, Nairobi).

In most cases, patients referred for ultrasound scan and other tests not only had to queue at the scanning room or lab, and make payments before they received the services, but they also had to navigate distances within the facility, a situation that further delayed their care process, as reported by one patient:

*So I went for scanning…initially, I was told to go and pay for the scan which was KES 1500 (*~*USD 15$), I paid for it and then I went for a scan, I queued there for a while then I was called and the scan was done* (38-year-old, married, hairdresser, urban, Nairobi).

In other facilities, participants simply could not get the scan because the ultrasound machine was broken, while others were sent elsewhere because the person-in-charge of doing the scan was absent. Some facilities were only providing ultrasound services at specific times and days of the week. Hence, patients who presented past working hours were referred for ultrasound services elsewhere. Even in cases where the ultrasound scan services were immediately available, some participants were unable to afford the service charge. Therefore, they had to postpone treatment.

Another reason for delays in receiving care was inadequate or lack of equipment and medical supplies. A participant from Nairobi waited for more than four days before being “washed” (removal of the retained products of conception) due to the lack of MVA equipment in the referral facility she visited:

*I came on Thursday evening and I was admitted, I was here on Friday, Saturday. On Sunday the doctor who did the rounds told me that there was no equipment to wash me (MVA) and that I should go to a different hospital; I told him whoever will come here on Monday on solve it, I will wait. So I relaxed and decided to wait for the equipment. They were brought on Monday … I was washed in the evening* (30-year-old, married, employed, urban, Nairobi).

Yet, this situation is not expected especially from a referral facility where most of the cases from primary-level are sent and there should be some guarantee of care.

### Patient-provider interactions

In addition to the waiting time, the quality of care for PAC services was also defined by the nature of interactions between providers and patients. Participants expressed their PAC experiences using constructs such as dignity and respect, autonomy, stigma and discrimination, privacy and confidentiality, and supportive care.

#### Dignity and respect

In-depth interviews with patients illustrated how the PAC provider attitudes reflected on patients’ experiences of dignity and respect when seeking PAC services. Most PAC patients interviewed across the seven counties agreed that service providers were friendly to them, as explained by one of the participants in Kajiado:

*A female tall and dark nurse, she really attended to me well. Even after she brought me here, she came back to check if I had been attended to. Yes, even the one we were with here attended to me well. You know some can attend to you, when you tell them to stop because you are in pain, they say you are disturbing and so on. But this one was saying sorry to me and attending to me well. I could tell him, wait a minute I feel sweaty and he could open the windows for me and attend to me well* (34-year-old, married, business woman, urban, Kajiado)*.*

PAC providers were considered as friendly by the participants for being empathetic, welcoming, checking on them, using encouraging words, sharing life experiences, and soothing and providing medication to ease pain. Other patients simply characterized provider friendliness based on how they greeted them, gave them medication at the right time, explained the procedures, and freely and openly answered their questions.

However, some participants reported cases of hostility, including physical and sexual abuse, threats, and providers speaking rudely or shouting, especially when they were suspected of inducing their abortion, or considered stubborn. Such experiences of rudeness are described by participants in Nairobi and Kajiado Counties who experienced unplanned pregnancies and were suspected of inducing their abortions:

*They have talked to me rudely, even when I go to the toilet, or even when it’s the needle that they put for me here that had come out, one of them quarreled with me* (24-years-old, married, housewife, urban, Nairobi).

*You know... not that they weren’t answering me alone, there were some who called them, they just came and answered them rudely. Some doctors are grumpy. There was one you called him, he asked, ‘what are you calling me for, stop disturbing me’, he just comes at his own time. So how he answers me, I just feel bad. We just kept quiet* (25-year-old, married, unemployed, urban, Kajiado).

The participants often felt vulnerable and helpless, and chose to endure in silence even when they were uncomfortable with the way they were being treated. Moreover, some participants reported that they had some questions, but felt unable to ask because of inherent fears that the providers might respond rudely or even stop the procedure:

*Because you may ask a doctor something and they start quarreling with you or telling you, “do not ask me questions”. So what will we do?* (37-year-old, married, farmer, urban, Kiambu).

*You know I feared them, I didn’t ask them. There was just one sister whom I asked and she told me that “you will just get pregnant and then I just kept quiet because I was satisfied with what she told me (22-year-old, married, business woman, urban, Nairobi).*

The few participants who were brave enough to ask questions or request more information on certain medications were threatened with dire consequences, which left them with the impression that healthcare providers are not receptive to questions from patients. This was especially evident in situations where the medications caused patients more pain instead of reducing it, as experienced by one of the participants in Laikipia:

*I told her that the injection was adding me more pain. She told me either you accept these drugs or go there. I asked her where? “won’t you die”, she said. I felt bad, because she didn’t want to tell me the reason for that medication* (28-year-old, separated, business woman, urban, Laikipia)*.*

Hostilities, such as cases of sexual harassment, were more commonly cited in one of the counties. Few participants recounted experiencing sexual abuses while at a particular health facility, yet, the issue remained unaddressed even after the facility administration was informed. During our facility observation, we interacted with patients complaining amongst themselves, describing how a particular male provider harassed them (requesting that they “hug him”) and threatened them. According to the participants, this situation seemed widespread and had persisted for some time. During the interviews, one of the participants recounted what she went through when one of the provider was “washing” her womb:

*When I was going to be washed I came and removed my pants…so I assumed that position, he came and saw no remains coming out but he started throwing hands at me. He asked me “you don’t feel well when you are touched to be ready for sex”… He touched my clit, and asked me “don’t you feel good to receive a man”… asked me whether I didn’t feel ready to have sex. … I felt very bad because, still that morning, the lady there came to my bed and told me she was also mistreated, the man touched her breasts or hugged her* (28-year-old, married, unemployed, urban, Kiambu).

Such situations demoralized participants, as they trusted the providers with their care. Whenever the patients refused the provider’ sexual advances, they would be neglected during treatment as reprisal. As such, some participants felt insecure and uncomfortable around male service providers, and wished male providers were transferred to male sections or other departments where there are no women.

*After I was washed, he came in and I didn’t feel secure because we were just the two of us…I didn’t fear a woman but when he came in, I didn’t like it. I was not impressed by him being in a woman’s job yet he is a man. He fondled my breast and I was not impressed…; I wouldn’t prefer being at the ward, his characters are not good, what he wanted to do to me yesterday night wasn't good to me. He tried to seduce me…he wanted to hold me but I moved away. I didn’t see his reason for having such a habit. In fact I preferred he be taken to the men section or maybe to a different department with no women* (20-year-old, separated, casual laborer, urban, Kiambu).

#### Privacy and confidentiality

The findings also revealed various issues around privacy and confidentiality of PAC services. For instance, in Kajiado and Garissa, most participants reported occasions where the treatment rooms were not secluded, thereby leading to other persons eavesdropping on the conversations between providers and patients or even popping into the room when the patient is naked on the table. Such invasion of privacy is described by one of the participants:

*...Because, of course, when you go to the theater you remove all clothes, so I was naked and there…the door was not closed, even those who were passing by could see you when you are being washed and there was no curtain. In fact that guy who came to clean just got me there naked* (25-year-old, married, unemployed, urban, Kajiado).

There were a few instances where providers spoke very loudly to the extent that other patients and/or bystanders could hear their conversation with patients, therefore leaving the latter embarrassed, as explained by a 23-year old young woman:

*He asked me “do you want to heal or what”, and they heard…Not good…Because you expect to tell the doctor your problems, not for the others to hear, it intimidated me* (23-yearold, single, urban, Kajiado).

This breach of privacy was aggravated when consultation or procedure rooms were close to the intake or waiting areas, or close to the washing rooms, or when patients interacted with providers in overcrowded places such as treatment or hospitalization rooms that had many patients. Other patients felt very uncomfortable when certain procedures like MVA were done with many healthcare providers in the treatment room. The fact that the procedure involved *“showing too much of one's private parts”* in the presence of many providers was seen as an embarrassing moment as expressed by one of the participants.

*Sometimes like being washed, because it’s more of you showing part of your body a lot, at some point, you feel they are too much in the room, I never loved that. You find that they are like eight people and like six of them are just storytelling, the students are not necessary there* (23-year-old, single, beautician, urban, Kiambu).

Although such privacy concerns were reported by many participants, it seemed more common among young and single women, some of whom had induced abortion, and therefore felt that the breach of confidentiality could expose them to stigma in their communities. Indeed, due to the emergency nature of care, most PAC patients would seek care at the health facilities closest to them, and as such, they felt that absence of confidentiality could breach their pregnancy and abortion experiences.

However, it is important to highlight that a few participants reported that some providers ensured the patients’ privacy by shutting the door during the process of care, drawing the curtains, and speaking in low tones. One particular patient reported how the provider handled interference as described by the quote below:

*You know, when you are in there and he talks to you, this other person will hear, the one who sits here. But the doctor didn’t want that. Whenever a person entered, he would tell him, “no, wait outside I treat this patient” So you enter after he finishes. He couldn’t let you enter when he is treating someone in there (30-year-old, married, business, rural, Migori).*

A 24-year-old participant interviewed in one of the referral facilities was very explicit on how she viewed PAC services provision that guarantees privacy. She remarked *“…doctors have work to do, he should be the only one with the patient in that room, people shouldn’t be opening and entering then”*. In her opinion, having only the doctor attending to the patient offers privacy for PAC patients.

#### Involvement in decision-making during care

Regarding involvement in the decisions around their care, virtually all participants reported that healthcare providers exclusively made the decision on treatment. Providers decided on the approach to treatment (i.e., choice between MVA and MA for the uterine evacuation) without consulting them or their caregivers:

*As I have told you, we are never in a position to tell the doctors how we want to be treated, you just come and you see them come to give you drugs* (23-year-old, single, urban, Kajiado).

Some participants were even surprised when the question about their involvement was asked, because to them, patients' non-involvement has always been the practice. Indeed, the shared perceptions that healthcare providers are more knowledgeable than patients pushed them to accept whichever medication was provided to them. One of the participants from Kajiado explains this:

*Mostly they do say that you cannot argue with the doctor, because he is the one who knows the medication to give you so that’s why you cannot always ask a question even when you have (33-year-old, married, employed, urban, Kajiado).*

As highlighted in the quotes, some participants chose to keep quiet even in situations where they felt they wanted to refuse certain procedures, as they feared backlash from providers. In rare cases, the healthcare providers would inform the patient about the treatment options and involve her and her caregivers in the decision-making.

#### *Treating them differently*: social support versus stigma and discrimination in the care process

Participants affirmed that they received supportive care demonstrated by providers’ empathy and administration of pain management drugs. Some patients, particularly those who had miscarriage and experienced psychological distress, described how providers took time to listen to their stories and advise them, as explained by two participants:

*Let me just say that they helped me, they have been very supportive. I don’t even know the name if the doctor or nurse who counseled me but, I felt they were good because when even the doctor counsels you, they must be very good because back then I was depressed and had pressure. But, they talked to you even on personal issues and they try to advise you not to do this and that.* (29-year-old, married, sales and marketing, urban, Laikipia).

*I really felt bad when I was told that the baby would be removed. They counseled me and told me that at least I have a child, some people don’t have any, and that encouraged me. And I was told to relax and also that I shouldn’t get pregnant very soon because then I would just have another abortion since the womb wasn't strong enough* (30-year-old, married, hairdresser, urban, Nairobi).

This counseling helped the participants to overcome the pain of pregnancy loss. In some cases, the psychological support received was reportedly detailed information on pain associated with procedures such as MVA. Getting such counseling helped participants to be calm and address their fears. One participant explained how she became psychologically prepared for the MVA and got rid of fears and misconceptions she had gathered from peers and the internet:

*They gave me enough information and they would prepare me. Like the MVA, I really feared, because I had heard stories, plus I was googling when I was in the ward because you are scared, you have been told it’s an incomplete abortion, you don’t even know what is that. So, when they realized I was scared, they first told me what it entails and everything. So, I think they helped me overcome it by giving me information, like trying to teach me what treatment they were giving me* (23-year old, single, beautician, urban, Kiambu).

It is noteworthy that patients who received such type of counseling were mostly those who reported respect and friendliness in their interactions with the providers. For patients suspected to have induced their abortions, their interactions with providers were largely characterized by tensions and rudeness devoid of supportive counseling. The young and/or unmarried women who were suspected of induced abortions were shouted at or spoken to rudely, sexually abused and threatened. In such cases, patients attributed the poor treatment to the fact that they had an induced abortion and their personal attributes (i.e., being young and single). This was further reflected in cases where PAC patients were persistently sexually harassed with no proper response from the facility administration. Patients therefore felt that providers were intentionally acting or behaving in certain disparaging ways because of their conditions. A 19-year-old single participant from one of the referral facilities in Nairobi County explained how she was mistreated right from the admission to the treatment area:

*When I got in there and was talking to the doctor, one of them was on Facebook just pressing the phone, another one was saying “I cannot do that is difficult”. Then a young doctor came and gave me this injection. Though, when he said he would help me, I waited for long. I was in so much pain and had to go to the bed all by myself. I cried but there was no doctor in sight. So, I just had to cry there and hang on and fortunately the baby came and I pushed, so when the baby came, that lady shouted “nurse come and help this girl”. That’s when she came and told me to push, and she went back and she came and checked if I had pushed… You know, I waited for long. I do see that when a patient comes here, they are attended to fast but here when you come you are just made to wait out there and feel the pain.* (19-yearold, single househelp, urban, Nairobi).

Nevertheless, there were few reported instances where providers protected PAC patients from community-driven stigma. During our health facility observations, a unique situation was noted in Kajiado County where a service provider was attempting to hide a PAC patient to protect both of them from community attack and humiliation because of having procured an abortion. In the follow-up discussion with the provider, he lamented the extreme levels of abortion-related stigma in the county including among PAC providers. Women are often forced to seek clandestine abortions and hide any complications, thus delaying seeking care. Providers also generally fear handling PAC patients. This fear is explained by the fact that community members (in most cases women) could raid the facility in a mob to attack and shame the patients and providers. According to one provider, this context forces most women who can afford to cross into Tanzania (across the border) to procure an abortion or seek treatment for post-abortion complications.

## Discussion

Our study findings offer critical insights into the quality of services women receive in Kenyan public health facilities following induced or spontaneous abortion-related complications such as bleeding, pain, or infection. Findings show that the patients’ choice of facility for PAC services was largely informed by participants’ previous experiences while seeking care for other ailments, regardless of whether the facility had the capacity to provide PAC services. Opinions were diverse on the waiting time before receiving care at the health facility with some participants reporting delays that made their care experience worse and their clinical conditions aggravated from mild or moderate to severe, while others justified the long waiting time with arguments around the large volume of patients and the limited number of health providers. It also emerged that the requirement for numerous tests and procedures such as obstetric or abdominal ultrasound scans prior to PAC treatment caused delays in care and increased the costs of care. This is despite studies emphasizing that trained providers (including nurses and midwives) can safely and effectively conduct uterine evacuation using medical abortion without the routine use of ultrasonography before or after abortion [18, 19].

Existing evidence shows that primary-level facilities have low capacity to handle post-abortion complications, and most of these facilities refer patients to high-level facilities [9]. Such structural weaknesses also impacted quality of care in terms of delays in accessing care and additional cost for patients. Indeed, the low capacity of facilities to support the referral processes (absence of fueled vehicles and ambulances), meant that patients had to facilitate their own transportation to the referral facility.

Recalling the domains and definition of person-centered care for abortion [16, 17], we identified gaps in the process and structure of abortion care that are fundamental components of quality of care. In general, participants indicated that healthcare providers were friendly with just a few reporting various forms of hostilities such as physical, emotional and sexual abuse, which engendered mistrust. At the same time, privacy and confidentiality were not guaranteed, while patients were seldom involved in the decision-making about their care. Yet, other studies have shown how patients’ involvement in decisions about their care, especially the choice of method to use for uterine evacuation (i.e., surgical procedure through the manual vacuum aspiration or medical evacuation procedure through misoprostol) contributes to mitigating abortion-related stigma and improving women's experiences with PAC [20]. The study by Cotter et al. in Nairobi and Kisumu in Kenya emphasized the key person-centered service indicators that could improve experiences of women while seeking PAC services in Kenya [17]. Key among these are trust, privacy and confidentiality, autonomy, communication and supportive care. In our current study, the patients sought post-abortion care after spontaneous or induced abortion and in most cases were in pain. There was a remarkable difference in care between patients who have had spontaneous abortion and those who have induced the abortion. Patients seeking care for spontaneous abortions received empathy from healthcare providers and psychosocial support to deal with the trauma. On the other hand, patients suspected to have had induced abortions reported stigma and discrimination, which worsened their care experiences. The environment of stigma and hostility occasionally led to delays in care seeking, being reserved and not revealing the full medical history to healthcare providers, or abstaining altogether from health facilities. Implementing training on the value clarification and attitude transformation could be very instrumental in improving the attitudes and behavior of providers involved in PAC provision.

This study is one of the rare studies that reported patients as victims of sexual abuse in health care setting within sub-Saharan Africa, contrasting with the various studies that present providers as victims [21, 22]. While a review conducted in 2002 identified sexual violence as one of the forms of violence experienced by women in health care settings, and called for more attention to the subject, very few enquiries have focused on this matter in the region, compared to the other forms of violence [23]. Moreover, studies that focused on the issue showed that even when reported, there seemed to be little or no consequence to the perpetrators of such malpractices within the health systems [24]. Likewise, our findings show that women, especially those who are vulnerable (young, poor, those who have induced abortions) seeking care in SSA health facilities are likely to face sexual harassment or abuse, and despite reporting, they were less likely to be trusted and action taken to redress the situation.

While participants’ conceptualization of “good” or “bad” care is primarily defined by the outcome of care (i.e., successful treatment, leaving the health facility in good or improved health condition), probed participants shared concerns about the care process including challenges in their interactions with providers or long waiting time. However, the outcome of care overrides the processes and structure of care such as hostility/abuse, absence of trained providers and PAC equipment leading to delayed care and lack of involvement of patients in the care process. Therefore, our findings show that there is a disconnect between what is actually quality of care as defined by the Donabedian conceptual model [16] and reported in other studies versus the patient definition of quality care which is often based on the outcome of care. And yet, subsequent care seeking pathways are largely informed by past experiences. While patients report “good care” when asked about their experiences focusing on the outcomes, the challenges experienced in the process of care might lead to delay in seeking services especially when faced with similar problems. Previous studies on care seeking found out how women perceived barriers in health facilities can lead to inadequate visits of health facilities for reproductive health care [25].

### Study limitations

While the paper builds on the process construct in the Donabedian’s model for evaluating quality of care to analyze the perspectives of women on the quality of PAC they received in Kenyan health facilities, this paper lacks the other complementary measures such as patient clinical outcomes (worsening of complication due to delayed or long waiting times, deaths and long term morbidities) and the structural status of health facilities (meaning how prepared the facilities were to offer PAC). Even so, this paper highlighted few structural elements of PAC within health facilities including the absence of PAC equipment, commodities and supplies, even though not in greater detail. In addition, since the patients interviewed were recruited at discharge (following completed care) and were describing their experiences retrospectively, there may have been some potential for recall bias. Lastly, given the sensitivity of the issue, potential influence of courtesy and social desirability bias might have influenced participants’ responses, as they might not have been at liberty to give negative feedback about the care they received.

## Conclusion

The gaps identified by the present study on the perspectives of women on quality of post-abortion services have far reaching implications on efforts to improve care seeking behaviors and well as how quality of abortion care can be assessed. Similarly, interventions that target to improve processes and structure of care such as staff training, availing equipment, commodities and supplies, need to be emphasized and modeled to improve patients' experiences at every stage of care and ultimately the overall quality of care. This implies the need to refine the tools and approaches for assessing women experiences of quality PAC to go far beyond the overriding clinical outcomes usually reported to integrate both structural and processes indicators of quality care. There is need for more research and evidence around quality of PAC in Kenya to prevent the adverse effects of unsafe abortion and facilitate improved health and wellbeing of women. Lastly, this paper also makes a call to action for actors in the health system to have candid discussions on sexual abuse perpetrated by providers and accompany such discussions with institutional, regulatory and clinical practice reforms. This may include clear guidelines on patient safeguarding and whistleblowing policies, quality of care to patients notwithstanding their health attribute, clear reporting channels known to patients, and strong consequences for such malpractices. Training of healthcare providers could also help to enhance awareness and prevention of sexual abuse in the process of care.

## Supplementary Information

**Additional file 1. **This a table presenting the interview guide that was used to interview the patients who received post-abortion care.

**Additional file 2. **This table presents the themes that emerged from the entire data set.

## Data Availability

The datasets used and/or analyzed during the current study are available from the corresponding author on reasonable request.
