# Supplementary material for: “They talked to me rudely”. Women perspectives on quality of post-abortion care in public health facilities in Kenya
Source: Reprod Health. 2023 Feb 27;20:35. doi: 10.1186/s12978-023-01580-5 (PMC9972787; doi:10.1186/s12978-023-01580-5)
Supplement: Supplementary file 1 — Additional file 1. This a table presenting the interview guide that was used to interview the patients who received post-abortion care. [file 12978_2023_1580_MOESM1_ESM.docx]

**Manuscript title:** “They talked to me rudely”. Women perspectives on quality of post-abortion care in public health facilities in Kenya

**Additional file 1:** Interview guide for women

| **Background of participants** | |
| --- | --- |
| Participants No. |  |
| Date of Interview |  |
| Name of interviewer |  |
| Study Site |  |
| Setting (Urban/Rural) |  |
| Age |  |
| Marital Status |  |
| Occupation |  |
| Remarks/ context |  |
| **Decision making to go for post-abortion care**: Please tell me a little about how you ended up in the hospital. | |
| How was the decision to visit the health facility for post-abortion care made? | Which signs prompted the decision? (Probe for excessive bleeding, shivers, foul smelling vaginal discharge, high fever) |
|  | How long did it take to make the decision (from the time you started experiencing the first symptoms to the time you decided to seek care)? |
|  | What and who influenced the decision? |
|  | Who was involved in making the decision to seek care? |
| How did you choose the facility to visit? (probe for: | Who made the decision? |
|  | Why did you choose this particular facility (good experience with previous visit, proximity, cost)? |
|  | Did you try anything home or elsewhere before visiting this facility? If yes, what was done? |
|  | Who took you here or called the ambulance? |
| **Post-abortion care process** | |
| Please describe the process through which you went once you arrived at the facility from the intake to the discharge. Probe for: | Where did you go first and what was done? |
|  | Time taken before receiving care |
|  | Exam and scan performed |
|  | Type of procedures used for washing/cleaning |
|  | Post-abortion counseling and contraceptives |
| **Patients experiences with post-abortion care services**: Now I want you to tell me about your notion of quality of care you received during this visit: | |
| Perception of good and bad care: In your conception and in your own words, what good care means for you? What about bad care? Probe for: | Which one did you receive during your visit? |
|  | What was different about this trip from your other visits for health care to the health facility? |
|  | What was you expecting in terms of care when arriving? What did you get? |
| Dignity and respect, communication and stigma and discrimination | Please, reflecting on the time it took you before being treated, what can you say about it? Probe:   - Short waiting time; long; just okay? - What is the reason that makes you think so about the time?   How would you describe the attitude of nurses, doctors and other health workers towards you during the care process? (Probe for:   - Friendly (talking nicely, supportive, etc.) - Hostile (insults, threats, spoke rudely, or physically abused) - Indifferent - Else |
|  | What would you say could have caused such behavior of healthcare providers? Probe for   - Type of abortion being treated (induced-spontaneous) - Marital status - Age... |
|  | Tell me about your experience talking to the providers (ex; asking questions you had about the kind of treatment you received) |
|  | What aspects of your communication with nurses, doctors and other staff did you like and which ones you did not like? |
|  | In your opinion, how did the nurses, doctors help you overcome any anxieties and fears you had prior to being treated? Probe:   - Pain management drugs - Empathic - Provision of information - No action (what could explain that?) |
|  | Did you, at any point of your journey within this facility, felt that you were treated differently from other patients?   - If yes, how differently were you handled? - What could be the reason? (probe: of your abortion, marital status or age for instance) |
| Autonomy: Can you tell me about how you were involved in the decisions about your care for instance the decision on which procedure to use for washing/cleaning the womb? | Who made decisions on how you were treated? (probe for only health workers, your companions, yourself) |
|  | How do you feel about being or not being involved in the decisions regarding your care? |
| Privacy and confidentiality | Thinking about the specific place in the facility where you were treated, what would you say about whether other people around could easily hear your conversations? |
|  | What would you say about the way our information was handled? |
|  | What are your views about the room where you were treated? Probe:   - Properly covered from the view of people not involved in your treatment - Your body was adequately covered |
|  | How about privacy from persons accompanying the provider or companions? |
| Cost and transparency of payment | How much did you pay for PAC (probe for:   - Direct cost within the facility (including consultation fees, treatments, drugs, tests, scan, etc.) - Indirect cost: transport, feedings, etc. |
|  | How was the payment done during your journey with the facility? Probe for   - Cashier or pharmacy with receipts - Informal payment directly to providers for treatment, medicine, or to fasten the care process etc. |
