# Supplementary material for: “They talked to me rudely”. Women perspectives on quality of post-abortion care in public health facilities in Kenya
Source: Reprod Health. 2023 Feb 27;20:35. doi: 10.1186/s12978-023-01580-5 (PMC9972787; doi:10.1186/s12978-023-01580-5)
Supplement: Supplementary file 2 — Additional file 2. This table presents the themes that emerged from the entire data set. [file 12978_2023_1580_MOESM2_ESM.docx]

**Manuscript title:** “They talked to me rudely”. Women perspectives on quality of post-abortion care in public health facilities in Kenya

**Additional File 2:** Emerging themes and sub-themes

| **Main Theme** | **Sub-theme 1** | **Sub-theme 2** |
| --- | --- | --- |
| Decision making process to seek PAC services | Pregnancy awareness | 1. Knew they were pregnant (test, pregancy sickness) 2. Did not know they were pregant |
|  | Abortion Related symptoms | 1. Excessive bleeding  2. Abdominal pains |
|  | The time frame for decisions to seek PAC services- | 1. Immediately went to the facility 2. Waited to see |
|  | Reasons for delays in seeking care | 1. It was not intense 2. Was hopping that it will reduce  3. Was waiting for husband and relative to accompany her 4. Was seeking money 5. Tried other types of care before visiting a facility |
| Care seeking pathways | Key players/actor to women’s care seeking pathways/decisions regarding where to seek PAC services- | 1. Woman herself 2. Partner/husband 3. Relatives |
|  | Type of care sought | 1. Health facilities (primary/referral) 2. Auto medication/pharmacy 3. Traditional healers |
|  | Reasons for chosing to seek PAC services from a particular health facility | 1. Referrals 2. Previous experiences with quality of care |
|  | Perception of quality care | 1. Good care (getting the right treatment, quality interraction with provider, short waiting time, cheap care) 2. Bad care (poor interraction with providers, not getting better, long waiting time, expensive care) |
|  | Reasons for referrals | 1. Lack of services needed (no equipment, no trained provider) 2. Could not afford the care cost |
| Getting to the facility and access to care from arrival | Means of transport | 1. Public transport (boda boda, Matatu, taxi) 2. Ambulance  3. On foot/personal transport |
|  | Time of arrival | 1. During day time 2. At night |
|  | Duration before being handled | 2. Handled immediately 3. Waited for more than 1 hour |
|  | Reasons for delay before being handled | 1. Overcrowding of patients 2. Provider availability/equipment availability 3. Payment or exams/scans before being attended to |
|  | Care delivery process | 1. Admission 2. seeing a provider 3. ultrasound scan and exams 4. Uterine evacuation |
|  | Post abortion contraception couselling and services | 1. No counseling 2. Counseling received |
|  | Reasons for not accessing contraceptives | 1. Preferred method not available 2. fear of husband/need of husband permission 3. Feer of side effects  4. Negative attitude of provider |
| Patients experience with the services at the health facilities (patient-rovider interactions) | Care received versus expected care | 1. Deferent from what was expected (bad or good) 2. Same as expected (bad or good) |
|  | Dignity and respect | 1. Good provider attitude (friendly/nice, supportive, presence of empathy) 2. Bad provider attitude (hostile, insulted, threatened, spoken rudely, verbal, sexual abuse, lack of empathy) |
|  | Patients autonomy/involvment regarding decisions made during treatment | 1. Patients or caregivers were not involved 2. Only health workers made decisions |
|  | Privacy and confidentiality | 1. Satisfied and conformable with privacy and confidentiality around the treatment area and treatment rooms (closed, private room, covered) 2. Not satisfied with treatment area/room (room not closed, crowded rooms, not covered, possible for other people to enter the room during treatment) |
|  | Provider and Patients communication during treatment | 1. Poor provider/patient communication (could not ask question or get answer to question, no information about medication or procedures performed)  2. Good provider/patient communication (possible to ask question and get responses, information about medication or procedures performed) |
|  | Social support and Supportive care from providers | 1. Supportive providers  2. Providers not supportive  3. Treated differently |
